# A comparative study of bone union and nonunion during distraction osteogenesis

**DOI:** 10.1186/s12891-022-06034-w

**Published:** 2022-12-03

**Authors:** Qi Liu, Ze Liu, Hongbin Guo, Min Wang, Jieyu Liang, Yi Zhang

**Affiliations:** 1 Department of Orthopaedics, Xiangya Hospital, Central South University, Hunan Province 410008 Changsha, China; 2 National Clinical Research Center for Geriatric Disorders, Xiangya Hospital, Central South University, Changsha, Hunan China; 3 Department of Endocrinology, Xiangya Hospital, Central South University, Changsha, Hunan Province China

**Keywords:** Bone union and nonunion, Distraction osteogenesis, External fixator, Pixel value ratio, Healing index, Lengthening index, External fixator index, Biochemical index

## Abstract

**Background:**

The clinical characteristics of bone nonunion during distraction osteogenesis (DO) were rarely discussed. This study was employed to specify the difference between bone union and nonunion during DO.

**Methods:**

The patients with bone lengthening were recruited in our study. The bone union cases indicated the ones that remove the external fixator successfully, whereas the bone nonunion represented the bridging callus did not appear even after 9 months (an absence of bridging callus for at least three out of four cortices on plain radiographs) that needs autogenous bone transplantation. The differences in the pixel value ratio (PVR) growth of regenerated callus, lengthening index (LI), healing index (HI), external fixation index (EFI) and blood biochemical indexes between bone union and nonunion were analyzed.

**Results:**

A total of 8 bone nonunion and 27 bone union subjects were included in this study. The PVR growth in bone nonunion was significantly lower than that in bone union (0.19 ± 0.06 vs. 0.32 ± 0.16, *P* = 0.048). Interestingly, the HI and EFI in bone nonunion was significantly higher than that in bone union (62.0 ± 31.4 vs. 37.0 ± 27.4, *P* = 0.036; 75.0 ± 30.9 vs. 49.9 ± 16.1, *P* = 0.006). However, no significant difference with regard to LI was identified (0.76 ± 0.52 vs. 0.77 ± 0.32, *P* = 0.976). Moreover, the circulating level of urea and lymphocyte count in bone union was significantly lower than that in bone nonunion (4.31 ± 1.05 vs. 5.17 ± 1.06, *P* = 0.049; 2.08 ± 0.67 vs. 2.73 ± 0.54, *P* = 0.018). On the contrary, the circulating level of magnesium in bone union was significantly higher than that in bone nonunion (0.87 ± 0.07 vs. 0.80 ± 0.07, *P* = 0.014).

**Conclusion:**

Compared to the bone union, the PVR growth was significantly lower, whereas the HI and EFI was significantly higher in the bone nonunion. Moreover, the circulating level of urea, magnesium and lymphocyte count was also different between these two. Therefore, the PVR, HI and EFI seems to be reliable and sensitive indicators to reflect the bone nonunion during DO, which might be considered in bone lengthening. Further prospective studies are still needed to elaborate the concerned issues.

**Supplementary Information:**

The online version contains supplementary material available at 10.1186/s12891-022-06034-w.

## Introduction

Distraction osteogenesis (DO) is a surgical technique that widely used to treat a variety of pathological conditions in children and adults, such as limbs length discrepancy, bone deformity or resection secondary to trauma, infection or malignant tumor [1]. The tension-stress rule of DO exerts continuous, stable and slow distraction force to living tissue, stimulates/activates tissue cell regeneration and growth, and promotes bone regeneration [2]. It has become an integral part of the arsenal in the orthopedics community worldwide, and the evolutionary development of the method has considerably improved the quality of life for millions around the world [3].

Meanwhile, the complications caused by DO may also need to be considered. Generally speaking, the DO-related complications include bone nonunion, new bone fracture, nail infection and relaxation, muscle contracture and joint stiffness, force line deviation, etc. [4–7]. Among them, the bone nonunion is a serious clinical issue that prolong the treatment period and increase the burden in bone lengthening [8]. However, the current evidence on bone nonunion is rather limited. Paley et al. [9] demonstrated that both technical (traumatic corticotomy, initial diastasis, instability, rapid distraction) and patient factors (infection, malnutrition, and metabolic) might lead to DO-related bone nonunion. Moreover, McKee et al. [10] found that the cigarette smoking was also associated with DO-related bone nonunion in limb lengthening. In addition, Liantis et al. [11] indicated that age, treatment days and fixator time were significantly correlated with a variety of DO-related complications, including bone nonunion. Papakostidis et al. [12] further suggested that the bone fracture risk will increase when the lengthening was larger than 8 cm. However, a comprehensive and systematic comparative study between bone union and nonunion during DO is still needed.

Several indicators were employed to assess the outcome in DO. Pixel value ratio (PVR) is mainly utilized to assess the maturity of late callus and the timing to remove the external fixator [13–17]. In addition, the lengthening index (LI), healing index (HI) and external fixator index (EFI) is served as general indicator to analysis bone healing during DO [18]. However, these above indicators were only considered in bone union cases. It was unclear if they could reflect the characteristics of bone nonunion. Moreover, some biochemical indexes are also reported to be associated with osteogenesis [19, 20]. Therefore, we aimed to investigate the difference in the following indexes between bone union and nonunion during DO: (1) the PVR growth pattern of regenerated callus; (2) the HI, LI and EFI; and (3) the comprehensive biochemical index, and identify some novel and sensitive indicators for bone nonunion during DO.

## Materials and methods

### Study design

This study was approved by the ethics committee of Xiangya Hospital of Central South University. The clinical and imaging data of patients who completed bone lengthening in Xiangya Hospital of Central South University were reviewed retrospectively. All surgical procedures were performed by the senior surgeons. The inclusion criteria were: (1) Lower limb lengthening by using Ilizarov technique; (2) Patients with bone union and nonunion during DO. Bone union indicated the ones that remove the external fixator successfully, whereas bone nonunion represented the bridging callus did not appear even after 9 months (an absence of bridging callus for at least three out of four cortices on plain radiographs) that needs autogenous bone transplantation[21–23].; (3) Primary surgery. The exclusion criteria were: (1) Amputation patients who are unable to complete bone lengthening therapy; (2) Patients with skeletal disorder affecting healing (e.g., congenital pseudarthrosis of tibia); (3) Patients with missing follow-up data. The Ilizarov technique was used for bone lengthening in femur and tibia. The distraction was initiated one week after the osteotomy (1.0 cm/day in juvenile and 0.75 cm/day in adult), and the patients were examined by X-ray monthly. The conditions to remove the external fixator were listed as follow: (1) bridging callus appears on three of the four cortices; (2) the fixation time is generally in line with the average extension index; (3) no abnormal feeling of weight-bearing after loosening the nut [24].

### The general characteristics of patients

A total of 27 bone union and 8 bone nonunion patients were recruited in our study. The general characteristics of patients including sex, age, BMI, lengthening length, cigarette smoking, alcohol drinking, external fixator type (unilateral or ring external fixator), reason for DO and site of osteotomy were collected, respectively.

### The difference in the PVR growth of regenerated callus

**Fig. 1:**
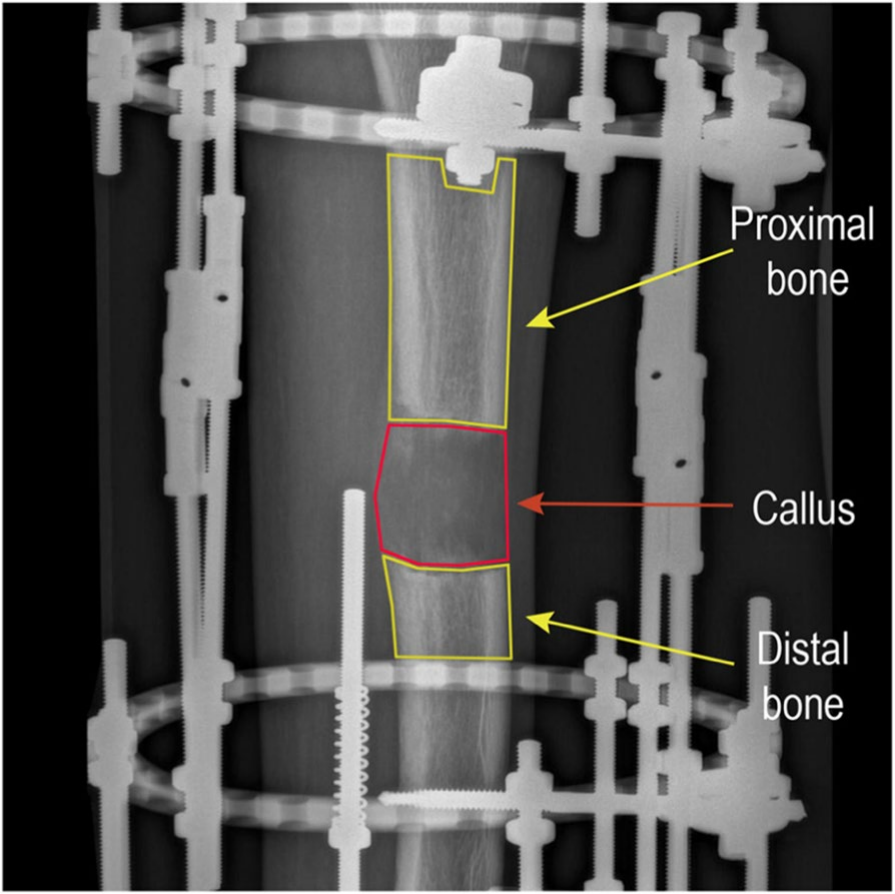
The assessment of PVR based on radiographic image

The PV of regenerated and adjacent bone after osteotomy were measured and recorded by using the image measurement tool of picture archiving the communication system (PACS) system (Fig. 1). All radiographs were taken by the same technician using the same equipment and were independently assessed by two senior orthopedists who were blinded to the subjects’ clinical symptoms. Subjects with inconsistent opinions were recalled after the survey and resolved by discussion. In order to improve the accuracy of PVR, the part of the metal bar was rigorously avoided. Then, the ratio of the regenerated callus PV to the adjacent bone PV was calculated. The higher PVR indicated that the regenerated callus was closer to the reference bone, whereas the lower PVR reflected a lower immaturity of the callus [17, 25]. The formula for the calculations of PVR were as follows:

$$\mathrm{PVR}=\frac{\text{Regenerated bone pixel value}}{\left(\text{Distal normal bone pixel value + Proximal normal bone pixel value}\right)\div2}$$

**Fig. 2:**
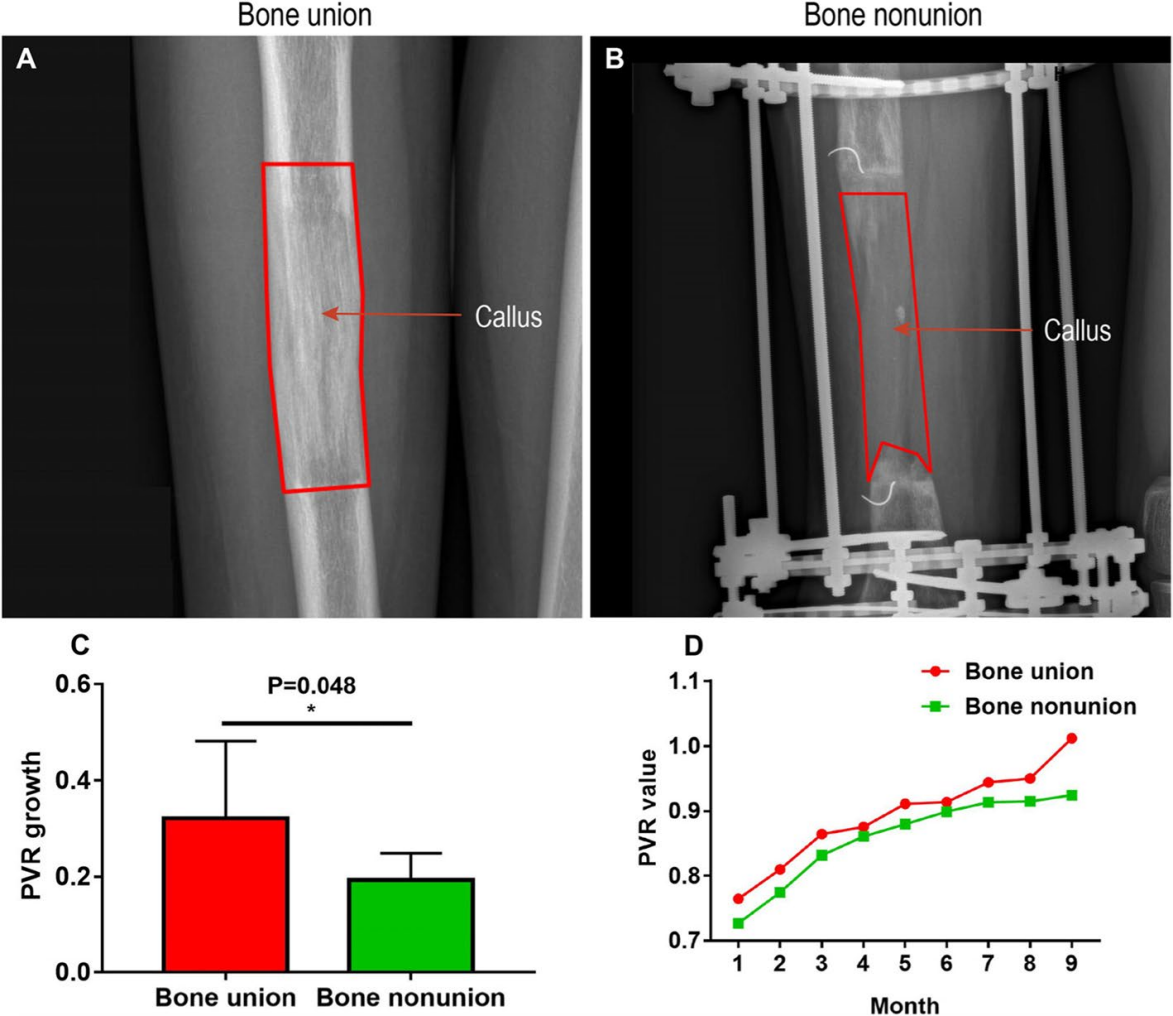
The difference in PVR growth pattern between bone union and nonunion during DO. **A** The representative image of bone union. **B** The representative image of bone nonunion. **C** The different PVR growth in bone union and nonunion. **D** The PVR growth pattern in bone union and nonunion

The PVR growth indicates the PVR difference between external fixator install and removal for bone union (Fig. 2A), or final autogenous bone transplantation for bone nonunion (Fig. 2B), respectively. The PVR growth was compared in bone union and nonunion. Moreover, the monthly PVR growth of regenerated callus in bone union and nonunion was also analyzed.

### The difference in healing index, lengthening index and external fixator index

The HI was calculated as the duration of complete consolidation (three cortices in distraction callus) in days divided by the length gained in centimeter, whereas the LI was the number of months required to achieve 1 cm lengthening [26, 27]. In addition, the EFI was calculated as dividing the using period of frames (days) by the distracted length of the bone (cm) [18]. All these three were decent and reliable indicators to reflect the bone healing potential, as well as the clinical outcome in DO. The HI, LI and EFI of the bone union and nonunion was therefore analyzed and compared.

### The difference in biochemical index

The comprehensive biochemical indexes before the surgery/osteotomy (CRP, total bilirubin, mean hemoglobin content, ESR, uric acid, urea, white blood cell count, lymphocyte count, basophil count, eosinophil count, fibrinogen, monocyte count, magnesium, calcium, phosphorus and total protein) in the bone union and nonunion was analyzed and compared.

### Statistical analysis

All the analyses were done by using the SPSS 26.0. The difference was analyzed by variance analysis. *P* < 0.05 was regarded as statistically significant.

## Results

### The general characteristics of patients

The general characteristics of patients were showed in Table 1. There was no significant statistical difference for sex, age, BMI, lengthening length, cigarette smoking, alcohol drinking, external fixator type, reason and site of osteotomy for DO between bone union and nonunion.

**Table 1:** The basic information between bone union and nonunion

	Bone union	Bone nonunion	*P* value
Sex			
Man	59.3%	62.5%	*P*=0.874
Woman	40.7%	37.5	
Age (years)	22.44±14.40	26.38±13.63	*P*=0.498
BMI	21.38±3.06	21.89±6.10	*P*=0.847
Lengthening length (cm)	6.94±3.41	8.96±4.86	*P*=0.195
Cigarette smoking			
Yes	3.7%	100%	*P*=0.594
No	96.3%	0.0%	
Alcohol drinking			
Yes	0.0%	12.5%	*P*=0.065
No	100%	87.5%	
External fixation type			
Unilateral	0.0%	87.5%	*P*=0.065
Ring	100%	12.5%	
Reason for DO			
Deformity	48.1%	87.5%	*P*=0.077
Trauma	25.9%	12.5%	
Tumor	7.4%	0	
Infection	18.6%	0	
Site of osteotomy			
Tibia	77.8%	50%	*P*=0.134
Femur	22.2%	50%	

### The PVR growth in bone nonunion was significantly lower

The PVR growth in bone nonunion was significantly lower than that in bone union during DO (0.19 ± 0.06 vs. 0.32 ± 0.16, *P* = 0.048) (Table 2; Fig. 2C). Obviously, the PVR grew more quickly in bone union (Supplementary Tables 1, Fig. 2D).

**Table 2:** The different PVR growth between bone union and nonunion

	Bone union (Mean ± SD)	Bone nonunion (Mean ± SD)	*P* value
PVR growth	0.32 ± 0.16	0.19 ± 0.06	***P* = 0.048**

### The healing index and external fixator index in bone nonunion was significantly higher

**Fig. 3:**
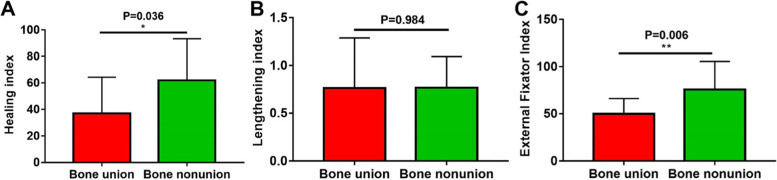
The comparison for HI, LI and EFI between bone union and nonunion during DO. **A** The difference in HI between bone union and nonunion. **B** The difference in LI between bone union and nonunion. **C** The difference in EFI between bone union and nonunion

The HI and EFI in bone nonunion was significantly higher than that in bone union (62.0 ± 31.4 vs. 37.0 ± 27.4, *P* = 0.036; 75.0 ± 30.9 vs. 49.9 ± 16.1, *P* = 0.006) (Table 3; Fig. 3A, C). However, no significant difference with regard to LI was identified (0.76 ± 0.52 vs. 0.77 ± 0.32, *P* = 0.976) (Table 3; Fig. 3B).

**Table 3:** The difference in HI, LI and EFI between bone union and nonunion

	Bone union (Mean ± SD)	Bone nonunion (Mean ± SD)	*P* value
HI	37.0 ± 27.4	62.0 ± 31.4	***P* = 0.036**
LI	0.76 ± 0.52	0.77 ± 0.32	*P* = 0.984
EFI	49.9 ± 16.1	75.0 ± 30.9	***P* = 0.006**

### The circulating level of urea, magnesium and lymphocyte count was different

The circulating level of urea and lymphocyte count in bone union was significantly lower (4.31 ± 1.05 vs. 5.17 ± 1.06, *P* = 0.049; 2.08 ± 0.67 vs. 2.73 ± 0.54, *P* = 0.018), whereas the circulating level of magnesium was significantly higher (0.87 ± 0.07 vs. 0.80 ± 0.07, *P* = 0.014) than that in bone nonunion (Table 4). With regard to other biochemical index, no significant difference was obtained (Supplementary Table 2).

**Table 4:** The difference in biochemical index between bone union and nonunion

	Bone union (Mean ± SD)	Bone nonunion (Mean ± SD)	*P* value
Magnesium (mmol/L)	0.87 ± 0.07	0.80 ± 0.07	***P* = 0.014**
Urea (mmol/L)	4.31 ± 1.05	5.17 ± 1.06	***P* = 0.049**
Lymphocyte count (×10^9/L)	2.08 ± 0.67	2.73 ± 0.54	***P* = 0.018**

## Discussions

**Fig. 4:**
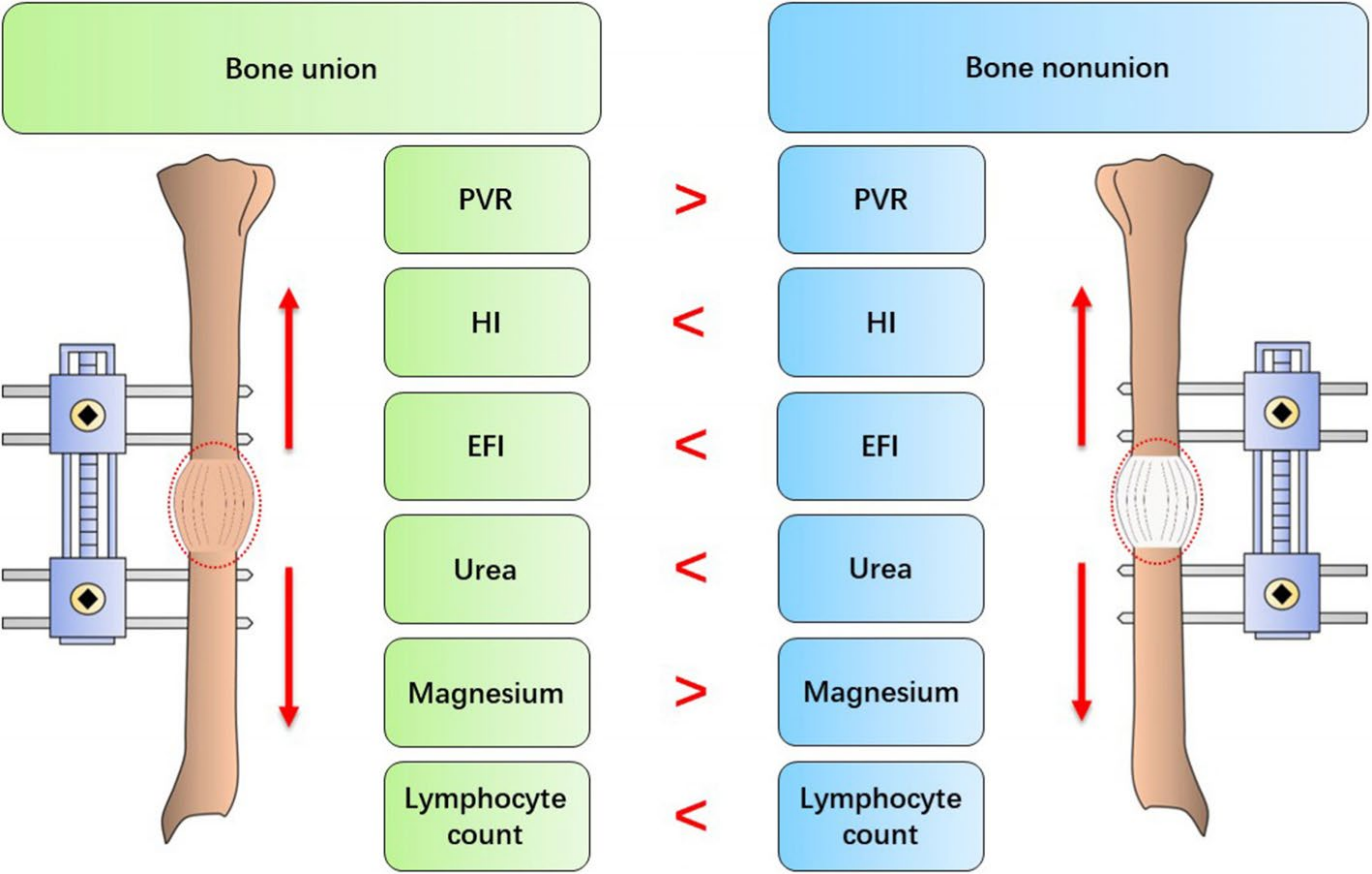
The schematic diagram for the results of this study

Compared to bone union, the PVR growth was significantly lower, whereas the HI and EFI was significantly higher in bone nonunion. The PVR, HI and EFI seems to be reliable and sensitive indicators to reflect the bone nonunion during DO. Moreover, the circulating level of urea, magnesium and lymphocyte count was also different between bone union and nonunion (Fig. 4).

To our best knowledge, the PVR has only been considered in bone union. Zhao et al. [17] suggested that the PVR could be severed as an objective measurement to guide the timing of external fixator removal (carry weight partially when the PVR of two cortices reached to 1, and carry weight fully when the PVR of three cortices reached to 1). Moreover, Bafor et al. [13] demonstrated that full weight-bearing could be initiated when the cortical PVR of 3/4 was at least 0.93 in bone lengthening. In addition, Vulcano et al. [16] indicated that a PVR value of 0.90 could be considered as bone healing. Zak et al. [28] further proved that the combination of PVR and subjective evaluation parameters (continuity, signal intensity and homogeneity of regenerated tissue) was conducive to monitoring the bone healing in DO. Futhermore, we previously found that the early PVR was gradually increasing in the first 3months after osteotomy, which might be significantly influenced by chronological age, sex, and lengthening site. Moreover, the early PVR seemed to be moderately inversely associated with HI and LI, respectively. In another word, the early PVR of callus may partly reflect the potential clinical outcome for DO [25]. On the basis of these above, our study further figured out the PVR growth pattern in bone nonunion during DO.

Interestingly, the HI and EFI in bone nonunion was significantly higher than that in bone union, whereas no significant difference with regard to LI was identified in our study. Although these three were reliable indicators for the outcome in DO [26, 27, 29–31], their difference should be identified and emphasized. Basically, LI refers to the average time to lengthen 1 cm. Therefore, the lengthening speed is similar between bone union and nonunion group, which ensures the homogeneity and comparability of the bone union and nonunion group (consistent average distraction speed). However, HI and EFI refers to the average time per 1 cm of bone healing or external fixator period, respectively. Indeed, the bone consolidation and healing in bone nonunion are exactly inferior to bone union. As a consequence, the HI and EFI is suggested to be routinely considered in bone lengthening.

Several biochemical indexes have been reported to be associated with bone disorder/osteogenesis before. Adunsky et al. [32] found that the urea might be served as a reliable indicator to predict the functional outcomes in hip fractures. In addition, Zhang et al. [33] found that magnesium could promote new bone formation in vivo, and Liu et al. [34] further demonstrated that magnesium could improve the osteogenic differentiation and angiogenesis in vitro. On the other hand, immune cells were considered to be important to bone dynamic balance [35]. Xu and Hong et al. [36, 37] indicated that the increased lymphocytes could lead to systemic bone loss by reducing the osteogenesis of bone marrow mesenchymal stem cells. Consistently, our results showed that the circulating level of urea, magnesium and lymphocyte count was different between bone union and nonunion. However, the circulating levels of magnesium and urea fall well within the normal ranges, and the differences could be related to the patients themselves. Therefore, more studies were still needed to address the issue.

The strengths of our study can be listed as follow: (1) this is the first comprehensive and systematic comparative study for bone union and nonunion during DO; (2) our results may be beneficial to the clinical management of bone lengthening. The limitations of the present study should also be acknowledged. First, several issues cannot be addressed due to the nature of retrospective study design. For example, the functional assessment during follow-up, the timing (midterm and final follow up) and variety (osteogenic index: osteocalcin, bone-specific alkaline phosphatase, etc.) of biochemical index cannot be considered in our study. Second, we cannot fully make sure that all the comparisons were on the same baseline. Third, the number of bone nonunion is relatively small, which may inevitability influence our results.

## Conclusion

Compared to bone union, the PVR growth was significantly lower, whereas the HI and EFI was significantly higher in bone nonunion. Moreover, the circulating level of urea, magnesium and lymphocyte count was also different between bone union and nonunion. Therefore, the PVR, HI and EFI seems to be reliable and sensitive indicators to reflect the bone nonunion during DO, which might be considered in bone lengthening. Further prospective studies are still needed to elaborate the concerned issues.

## Supplementary Information

**Additional file 1: Supplementary Table 1.** The different PVR growth pattern between bone union and nonunion. **Supplementary Table 2.** The difference in biochemical index between bone union and nonunion.

## Data Availability

The datasets used and/or analyzed during the current study are available from the corresponding author on reasonable request.
