# Supplementary material for: A comparative study of bone union and nonunion during distraction osteogenesis
Source: BMC Musculoskelet Disord. 2022 Dec 3;23:1053. doi: 10.1186/s12891-022-06034-w (PMC9719176; doi:10.1186/s12891-022-06034-w)
Supplement: Supplementary file 1 — Additional file 1: Supplementary Table 1. The different PVR growth pattern between bone union and nonunion. Supplementary Table 2. The difference in biochemical index between bone union and nonunion. [file 12891_2022_6034_MOESM1_ESM.docx]

Supplementary Table 1 The different PVR growth pattern between bone union and nonunion

|  | Bone union  (Mean±SD) | Bone nonunion  (Mean±SD) | P value |
| --- | --- | --- | --- |
| First month | 0.76±0.07 | 0.72±0.06 | P=0.168 |
| Second month | 0.81±0.09 | 0.77±0.05 | P=0.341 |
| Third month | 0.86±0.07 | 0.83±0.06 | P=0.351 |
| Forth month | 0.88±0.11 | 0.86±0.04 | P=0.730 |
| Fifth month | 0.91±0.10 | 0.88±0.09 | P=0.499 |
| Sixth month | 0.91±0.11 | 0.90±0.07 | P=0.784 |
| Seventh month | 0.94±0.13 | 0.91±0.37 | P=0.652 |
| Eighth month | 0.95±0.16 | 0.91±0.09 | P=0.684 |
| Ninth month | 1.01±0.20 | 0.92±0.04 | P=0.349 |

Supplementary Table 2 The difference in biochemical index between bone union and nonunion

|  | Bone union  (Mean±SD) | Bone nonunion  (Mean±SD) | P value |
| --- | --- | --- | --- |
| CRP (mg/L) | 4.24±5.36 | 1.31±0.34 | P=0.244 |
| Total bilirubin (μmol/L) | 10.04±5.35 | 10.54±6.69 | P=0.830 |
| Mean hemoglobin content (g/L) | 28.55±3.63 | 29.60±1.56 | P=0.465 |
| ESR (mm/h) | 26.31±22.50 | 9.00±9.08 | P=0.086 |
| Uric acid (μmol/L) | 322.50±89.69 | 325.49±71.19 | P=0.932 |
| White blood cell count (×10^9/L) | 6.47±1.35 | 6.54±1.01 | P=0.903 |
| Basophil count (×10^9/L) | 0.02±0.04 | 0.02±0.04 | P=0.422 |
| Eosinophil count (×10^9/L) | 0.35±0.70 | 0.15±0.12 | P=0.967 |
| Fibrinogen (g/L) | 2.81±0.82 | 2.85±0.89 | P=0.914 |
| Monocyte count (×10^9/L) | 0.51±0.16 | 0.50±0.15 | P=0.820 |
| Calcium (mmol/L) | 2.34±0.11 | 2.36±0.16 | P=0.610 |
| Phosphorus (mmol/L) | 1.43±0.40 | 1.44±0.24 | P=0.944 |
| Total protein (g/L) | 68.81±5.43 | 69.81±4.81 | P=0.642 |
